# Challenges in Fabrication of Tissue-Engineered Cartilage with Correct Cellular Colonization and Extracellular Matrix Assembly

**DOI:** 10.3390/ijms19092700

**Published:** 2018-09-11

**Authors:** Mikko J. Lammi, Juha Piltti, Juha Prittinen, Chengjuan Qu

**Affiliations:** 1 Key Laboratory of Trace Elements and Endemic Diseases, National Health and Family Planning, Institute of Endemic Diseases, School of Public Health of Health Science Center, Xi’an Jiaotong University, Xi’an 710061, China; 2 Department of Integrative Medical Biology, University of Umeå, 901 87 Umeå, Sweden; juha.piltti@umu.se (J.P.); juha.prittinen@umu.se (J.P.); chengjuan.qu@umu.se (C.Q.); 3 Nordlab Kokkola, Keski-Pohjanmaa Central Hospital Soite, 40620 Kokkola, Finland

**Keywords:** articular cartilage, tissue engineering, cell colonization, extracellular matrix, cartilage architecture

## Abstract

A correct articular cartilage ultrastructure regarding its structural components and cellularity is important for appropriate performance of tissue-engineered articular cartilage. Various scaffold-based, as well as scaffold-free, culture models have been under development to manufacture functional cartilage tissue. Even decellularized tissues have been considered as a potential choice for cellular seeding and tissue fabrication. Pore size, interconnectivity, and functionalization of the scaffold architecture can be varied. Increased mechanical function requires a dense scaffold, which also easily restricts cellular access within the scaffold at seeding. High pore size enhances nutrient transport, while small pore size improves cellular interactions and scaffold resorption. In scaffold-free cultures, the cells assemble the tissue completely by themselves; in optimized cultures, they should be able to fabricate native-like tissue. Decellularized cartilage has a native ultrastructure, although it is a challenge to obtain proper cellular colonization during cell seeding. Bioprinting can, in principle, provide the tissue with correct cellularity and extracellular matrix content, although it is still an open question as to how the correct molecular interaction and structure of extracellular matrix could be achieved. These are challenges facing the ongoing efforts to manufacture optimal articular cartilage.

## 1. Introduction

During our daily activities, body movements predispose our bones to high mechanical loads, which are dampened by the articular cartilage at the bone ends. Although the joint structure makes almost frictionless movement of the bones possible, a progressive degeneration of the cartilage tissue often occurs, especially in the ageing cartilage. A degeneration of cartilage can develop due to trauma, which leads to focal chondral or osteochondral defects, or due to a diffuse loss of the cartilage in a generalized disease, such as osteoarthritis (OA) [1]. Surgical joint replacements are a well-established means to treat advanced-stage cartilage defects, although there has been considerable interest in cell-based repair techniques for the traumatic and early stage OA as well. The first human autologous chondrocyte implantations to repair the cartilage defects of the knee were reported as early as the 1990s [2].

In another technique called cartilage mosaicplasty or osteochondral autograft transfer system, osteochondral cylinders (4–10 mm in diameter) are implanted into the lesion holes in the damaged area [3]. A fibrin clot, which forms between the cylinders, will then give rise to fibrocartilage connecting the adjacent implanted cylinders. A problem with this technique is the difficulty to restore the shape of the cartilage surface, a poor integration of the implanted cylinders to the adjacent cartilage and, naturally, new lesion sites produced at the site of the removed tissue [4]. 

Since the injected chondrocyte suspensions used for cartilage repair form a soft implant, it takes a long time for the repair tissue to produce a sufficient amount of extracellular matrix (ECM) that would give the cartilage good and enduring biomechanical properties [5]. This also makes the recovery time long for the patients [5]. Therefore, one approach to obtain an implantable construct is to grow a cell-based tissue-engineered cartilage under laboratory conditions using scaffolds [6] or even scaffold-free culture systems. The tissue-engineered osteochondral implants, which resemble native articular cartilage in their biomechanical properties and function, would be useful to replace tissues needed for the mosaicplasty technique of cartilage repair.

During the last two decades, promising new strategies that use assorted scaffolds and cell sources to induce chondrocyte regeneration have emerged. A variety of scaffold and cell combinations have been under investigation [7]. Synthesized or purified natural biomaterial scaffolds can provide a structural basis for cartilage repair and stimulate the healing processes of damaged tissues. Scaffold-free constructs, as well as decellularized and bioprinted tissues, also offer potential alternatives for cartilage tissue engineering.

## 2. Scaffolds for Cartilage Tissue Engineering

There are many different factors that contribute to the feasibility of scaffold materials for cartilage and bone tissue engineering [8]. A successful biodegradable polymer should allow good cellular colonization and growth of the chondrocytes or the differentiated stem cells, and it should not show toxic or inflammatory responses when implanted. It also has sufficiently high porosity to provide a good interconnectivity as well as a large surface area and an adequate space for the ECM to assemble. Optimally, it will also totally degrade with a controllable resorption rate, first supporting the repair cells and tissues but later allowing space for the forming repair neotissue. Besides, the polymer should be reproducibly processable into three-dimensional shapes. Since chondrocytes also easily lose their phenotype in monolayer cultures [9], conditions maintaining or inducing the chondrogenic phenotype are of utmost importance, especially when implanting stem cells.

The scaffolds can either be natural or synthetic polymers or a combination of the two. Both forms have their advantages and disadvantages. Natural polymers are normally better suited for the repair of relatively small lesions, while there are versatile manufacturing methods today to tailor the properties of synthetic polymers, such as their improved mechanical properties [10]. More reliable sources of raw materials used to manufacture synthetic polymers also reduce the risks of immunogenicity [11]. 

### 2.1. Synthetic Scaffold Materials

Synthetic biodegradable polymers have been widely investigated as orthopedic devices and scaffolds [12]. The first synthetic polymers used for chondrocyte co-cultures were either polyglycolic or polylactic acid scaffolds or their copolymers [13,14,15]. Since then, many other materials have also been considered as scaffold materials for cartilage repair, such as polycaprolactone [16], poly(vinylalcohol) [17], and various other (co)polymers [18]. They can also be used together with natural biomaterials. Moreover, plans to manufacture biphasic tissue-engineered constructs that combine cartilage and bone components of collagen and hydroxyapatite have already been introduced in 2004 [19]. 

Considering the colonization of seeded cells, synthetic materials used as scaffolds often have well-defined structural assembly (for instance, a knitted structure), which gives a well-organized form for the scaffold material. The porosity and interconnectivity can also be regulated in a controlled way, which is important for proper embedding and colonization of cells within the scaffold. In general, three-dimensional scaffold/tissue constructs should be such that vascularization would ensure a good cellular viability by providing the necessary delivery of nutrients and oxygen and removal of metabolic waste products. A capillary ingrowth may not always be adequately obtained; thus, the use of interconnected macropore structure of 300–500 µm can enhance the diffusion of nutrients and metabolic by-products in and out of the scaffold, although their transportation may still not be sufficient for large constructs [20]. However, the cartilage is an exceptional tissue that is completely dependent on the diffusion of nutrients and by-products due to lack of vasculature in the tissue; therefore, the clearance of possibly harmful by-products may be more important than the nutritional aspect.

**Figure 1:**
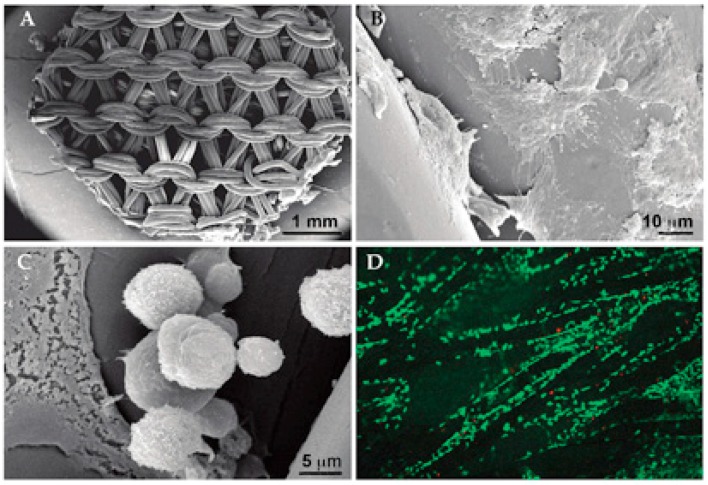
Chondrocyte attachment on the knitted poly-l,d-lactic acid scaffold. (**A**) A knitted scaffold used for the cellular embedding. (**B**) A high proportion of the seeded chondrocytes (72%) adhered on the surface of the scaffold fibers within 12 h of cellular seeding, but most of the cells spread and flattened on the material after the initial adhesion. (**C**) Some chondrocytes could still adopt a spherical morphology. (**D**) Live/dead staining showed a good viability of the chondrocytes (green cells); however, the cellular attachment mainly occurred on scaffold fibrils, leaving most of the space unoccupied by the cells. Some red-stained dead cells were visible.

Nonetheless, manufacturing techniques that produce large open spaces in cellular dimensions do not easily allow full cellular colonization of the chondrocytes upon cell embedding. An example of this is a knitted poly-l,d-lactic acid scaffold (Figure 1A), which has large open areas not covered by the scaffold. In our studies, when the chondrocytes were embedded within the mesh for 12 h, the cells obviously sensed the material surface as a two-dimensional environment and mostly adapted a flattened cellular morphology (Figure 1B), similar to the chondrocyte behavior in monolayers on cell culture plastic. However, some cells had a spheroidal morphology, indicating the preserved phenotype (Figure 1C). A confocal image taken after live/dead staining clearly showed the attachment of the cells mainly on the biomaterial surface, while the cells did not occupy the open spaces of the construct (Figure 1D) [21].

Biodegradation of polylactic and polyglycolic acid occurs by hydrolytic scission and nonspecific enzymatic action, which releases lactic and glycolic acids, respectively [22]. Although these degradation product can be easily metabolized by the body, they produce an acidic environment at the site of the implant [23,24] that may be toxic to the cells, thereby limiting their clinical usefulness to some extent. In this context, it must be kept in mind that chondrocytes in the cartilage live under physiologically acidic environment and are relatively resistant to pH changes. Nevertheless, incorporation of basic salts within the polylactic acid–polyglycolic acid implants has been shown to be effective in controlling pH changes resulting from the biodegradation of the material [25]. Local inflammatory reaction has also been reported for polyesters [26]. However, different types of polyesters can nowadays be utilized to modify the mechanical strength and degradation rates of these biomaterials [10].

A failure of the implant due to the intrinsic properties of the scaffold may also lead to a loss of the desired cells at the site of repair. For instance, too-high stiffness of the poly-l,d-lactic acid (96:4) was found to lead to a delamination of the implant in goat knee joint after implantation [27], while in young pigs, the same scaffolds were observed to have embedded inside femoral bone [21]. The failure in the goat was interpreted to be due to the thin cartilage and hard subchondral bone, which obviously jeopardized proper fixation of the implant despite the fact that sutures were used to immobilize it. In young pigs, the stiffness of the implant apparently caused repeated microfractures of the subchondral bone, weakening it so that the implant could penetrate into the bone.

### 2.2. Natural Scaffold Materials

Large macromolecules that are synthesized and secreted by the chondrocytes assemble a specific architecture of the articular cartilage ECM. These include type II collagen, hyaluronan, and aggrecan. They are also the obvious choice to be considered as scaffolding biomaterials for cartilage repair purposes. Other natural macromolecules, such as chitosan and alginate, have also attracted wide interest.

#### 2.2.1. Collagen as a Scaffold for Cells

Collagens are native fibrous structures of the connective tissues. The triple helices of collagens form nanosize fibrils (about 300 nm in length), which are in soluble form in mild hydrochloric or acetic acid solutions. Upon neutralization, the collagens will form gels. The most common collagen type available is type I collagen, while type II collagen—the major collagen type in cartilage—is not easily available. Nevertheless, recombinant human type II collagen has been available for in vitro tissue engineering, and even experimental cartilage repair purposes [28,29,30,31], although it has not been used very widely due to limited availability.

**Figure 2:**
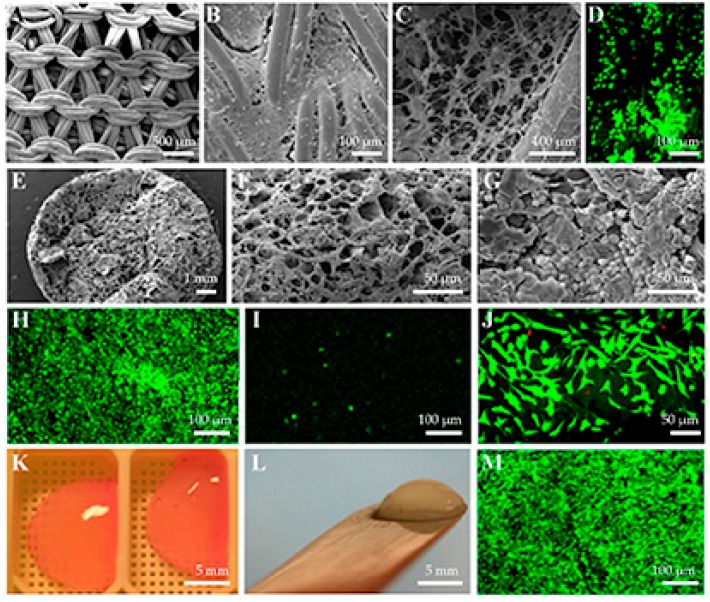
Examples of the use of recombinant human type II collagen as a biomaterial for primary chondrocytes. (**A**) The nonseeded knitted poly-l,d-lactic acid disc. (**B**) The surface of knitted poly-l,d-lactic acid disc filled with cross-linked recombinant human type II collagen seeded with primary chondrocytes. (**C**) The inner part of knitted poly-l,d-lactic acid disc filled with cross-linked recombinant human type II collagen seeded with primary chondrocytes. (**D**) The inner part of knitted poly-l,d-lactic acid disc filled with cross-linked recombinant human type II collagen seeded with primary chondrocytes and cultured with live/dead fluorochromes to visualize the live (green) and dead cells (red). (**E**) Recombinant human type II collagen sponge shows the porous structure of the material. (**F**) The porosity of recombinant human type II collagen sponge is also obvious inside the material. (**G**) The chondrocytes adhere well to the surface of recombinant human type II collagen sponge. (**H**) Live/dead staining indicates good cell viability on the surface of recombinant human type II collagen sponge. (**I**) However, only a few chondrocytes can reach the most inner part of recombinant human type II collagen sponge. (**J**) The chondrocytes seeded on the surface of recombinant human type II collagen membrane adhere well but show fibroblastic shapes and apparently dedifferentiated phenotype. (**K**) The chondrocytes mixed with soluble recombinant human type II collagen form the cell-embedded gels. (**L**) The chondrocyte seeded recombinant human type II collagen gels also stiffen during a two-week culture period. (**M**) The chondrocytes embedded in recombinant human type II collagen also have good cell viability for at least four weeks, as shown by live/dead staining.

Soluble collagen can be used in various ways to provide cells a supporting scaffold. One way is to soak the synthetic scaffold, for instance made of poly-l,d-lactic acid (Figure 2A), in acidic collagen solution and crosslink it after neutralization [28]. The lyophilized material can then be used for cellular seeding (Figure 2B,C) [28]. The chondrocytes cultured in such a scaffold have good colonization and viability (Figure 2D) [28]. Collagen can also be used to prepare collagen sponges, which—on scanning electron microscopic inspection—affirm a nice porous structure not only on the surface of the construct (Figure 2E) but also in the middle of the material (Figure 2F). However, cellular seeding reveals the challenge in the preparation of the scaffold. Although both scanning electron microscopic (Figure 2G) and live/dead staining (Figure 2H) show good cellular colonization on the surface of the material [28], an image taken from the middle of the sponge (Figure 2I) indicates that only a few chondrocytes were able to reach the middle part of the construct. This clearly shows that besides material porosity, the interconnectivity of the pores is also of utmost importance for colonizing cells to avoid the dead ends of the material.

The principle of using membranous sheets has been considered as one way to construct scaffolds for tissue engineering purposes. The cells can then be cultured on the membranes. Although it is possible to make membranes from collagen, it is important to use a technique that would provide some porosity for the structure; otherwise, the chondrocytes may adopt the flattened morphology typical for their growth on the cell culture plastic (Figure 2J) [28]. Mixtures of collagen and chondrocyte suspension can be exploited to obtain cell-embedded gels (Figure 2K), which can be easily handled (Figure 2L). This technology also provides a good cellular colonization within the construct (Figure 2M) [29].

#### 2.2.2. Chitosan as a Scaffold for Cells

Chitosan is a linear and cationic carbohydrate polymer, which consists of repeating units of β(1–4)-linked d-glucosamine and *N*-acetyl-d-glucosamine [32]. A commercial production of chitosan is based on alkaline deacetylation of chitin, which is typically derived from crustaceans, such as crabs and shrimps, or from cell walls of fungi or insects [33]. Depending on the manufacturing process, the chemical properties, such as molecular weight and deacetylation degree of chitosan polymer, can be modified [34]. The typical molecular weight of commercially produced chitosan polymer is in the range of 300–1000 kDa, and the deacetylation degree is in the range of 50–95%. Both the molecular weight and deacetylation degree have an influence on viscosity and porosity of chitosan-based polymers. Pore sizes and their interconnected orientation have a significant role in the mechanical properties and the cellular colonization of the chitosan polymer. Furthermore, the functional amino and hydroxyl groups of chitosan polymer can be modified, and this may influence, for example, the mechanical properties and reactivity of chitosan. The deacetylation degree is a critical factor because it has a connection to all the chemical, physical, and biological features of chitosan and chitosan-derived materials [35]. The degree of deacetylation is proportional to the chitosan’s degree of crystallinity. Nowadays, many different kinds of techniques can be used to produce porous chitosan scaffolding materials [33,36].

Chitosan is a promising material for cartilage tissue engineering purposes because it has both good biocompatibility and biodegradability. Additionally, chitosan and its degradation products are nontoxic, it is nonimmunogenic and possesses antimicrobial activity, and its cationic nature favors formation of complexes with negatively charged glycosaminoglycans (GAGs), proteoglycans (PGs), and other anionic compounds [34,37,38]. The good biodegradability of chitosan is related to its chemical structure. Hydrolytic enzymes, such as lysozymes, can degrade chitosan in in vivo conditions. The rate of the biodegradation process is dependent on the deacetylation degree, and the basic rule is that the higher the deacetylation degree, the slower is the degradation process [35]. In addition to the deacetylation degree, there are also additional factors, such as molecular weight and the degree of crystallinity and water content, which have an influence on chitosan degradation.

Chitosan can maintain both chondrocytic cell morphology and chondrocyte phenotype-specific ECM production and decrease certain catabolic responses [39,40,41]. The mechanism is at least partly related to interactions of chitosan with the native articular cartilage-related GAGs and the hyaluronan [33]. Due to its chemical resemblance with the GAGs and hyaluronan composition, it has a molecular interaction to ease the maintenance of the articular cartilage [39]. A modification of chitosan polymer is also possible by combining or blending it with other natural polymers or synthetic polymers [42]. Additionally, minerals, such as calcium phosphate and hydroxyapatite, can be mixed with it. These kinds of composites are fabricated to improve or modify, for example, the mechanical strength or the porosity of chitosan scaffolds. For implanting cells in the desired location, they can be seeded in porous chitosan matrices or the cells can be mixed with chitosan gel, which is then injected into the cartilage lesion site [33,43].

##### Chitosan Hydrogels

One of the first chitosan cell implantation applications designed for articular cartilage repair was based on the gels formed when chondrocytes were mixed with chitosan and glycerol-phosphate disodium salt solution [43]. The mixture of the cells and autogelling chitosan solution turned into a gel implant after the injection, immobilizing the cells at the desired density within the implant. After a three-week monitoring period, the implanted chondrocytes expressed normal ECM components [43]. Another in vivo study showed that chitosan hydrogel maintained living cells and chondrocyte phenotype-specific ECM production in in vivo conditions [44]. Chitosan hydrogel provided mechanical support to the injured cartilage area, and the gel scaffold was shown to reside in a treated area for at least one week after the implantations in animals. However, chitosan gelling happens slowly, and there is therefore a risk that the gel solution can leak out from a joint cavity and induce cartilage-like repair tissue formation in wrong places [45].

BST-CarGel^®^ (Smith & Nephew) is a commercial chitosan and glycerol phosphate-based hydrogel scaffold, which is used clinically with the microfracture method to treat cartilage defects. The manufacturing process of BST-CarGel^®^ is done at a physiological pH, and both the optimal pH and the composition of the BST-CarGel^®^ scaffold support the formation of stable blood clots and the cartilage repair tissue formation into a microdrilled area [46]. The blood entering the drilled area enables, for instance, the colonization of osteogenesis- and chondrogenesis-inducing mesenchymal stem cells (MSCs) into the microdrilled area. Clinical follow-up studies have revealed that the BST-CarGel^®^ material improves structural, cellular, and clinical outcome of these operations in comparison to traditional microfracture methods that are performed without the support of a BST-CarGel^®^ scaffold [47,48,49,50].

Chitosan composite hydrogels are developed to improve tissue engineering properties and avoid drawbacks of the basic chitosan hydrogels [42]. The native cartilage ECM components, such as type II collagen or chondroitin sulfate, can be incorporated into chitosan hydrogel matrixes. The incorporation of type II collagen has especially been shown to induce chondrogenesis in chondrocytes cultured in three-dimensional chitosan hydrogel matrix [51]. Chondrocytes and MSCs have specific ECM receptors—integrins—that ease the colonization and adhesion of the cells within the ECM. Despite the promising results, long-term clinical chitosan hydrogels studies will be needed to evaluate safety and clinical applicability. In addition to natural polymers, chitosan composites have been made with synthetic polymers, such as polycaprolactone and polylactic acid [42,52]. In addition, a blend of chitosan and β-chitin has been used to form hydrogel and sponge-like scaffold for chondrocyte culturing [53].

##### Preformed Chitosan Structures

Chitosan scaffolds can be manufactured as preformed structures instead of injectable hydrogel solutions. A hydrothermally cross-linked chitosan scaffold is a better material for seeded chondrocytes to produce both mechanically and biologically promising constructs for cartilage tissue engineering compared to an uncross-linked one [54]. Importantly, short-lasting experiments have revealed induction of chondrocyte proliferation and the maintenance of spherical cell morphology; however, further experiments will be needed to evaluate its clinical usability. In addition to the mechanical support for tissues and cells, the preformed chitosan scaffolds can also be used to release additional factors, such as growth factors, to induce chondrogenic cell responses. Mixed chitosan-platelet-rich plasma scaffolds have been tested for bone, wound healing, and cartilage regeneration processes [55]. They supported the differentiation of human chondrocytes and the expression of type II collagen, which is important for the native-like hyaline cartilage production.

Transforming growth factor beta 1 (TGF-β_1_) is a growth factor that stimulates chondrocyte cell proliferation, their phenotype-specific ECM formation, and inhibits terminal differentiation of the chondrocytes [56]. These features make it an interesting factor for tissue engineering applications and cellular colonization as it can be loaded to the chitosan scaffolds using different strategies. TGF-β_1_ loaded on chitosan microspheres with emulsification method can be utilized to increase the level of TGF-β_1_ in tissue [57]. TGF-β_1_ levels can also be increased with chitosan loaded with the TGF-β_1_ encoding plasmid [58]. Both strategies have been shown to promote chondrocyte phenotype-specific responses in short-term in vitro studies. Notably, another gene-activated matrix application, which was based on the use of hyaluronan and chitosan nanoparticles embedded with TGF-β_1_ encoding plasmid DNA, was found to induce the TGF-β_1_ expression in chondrocytes and to increase their cellular proliferation, although an overall effect to the phenotype-specific ECM production was not observed [59]. 

Novel porous and sponge-like chitosan composite materials have been developed to improve the existing chitosan matrixes. Although chitosan has many beneficial properties, further optimizations of the mechanical strength, the structures of pores, and the biodegradability of chitosan scaffolds by blending it with some other polymer would be often desirable. Chitosan can be cross-linked to natural and synthetic polymers ionically and covalently [60,61]. The desired cross-linked final products can be achieved by chemical methods, while some cross-linkers, such as glutaraldehyde, have cytotoxicity risks related both to unreacted cross-linking chemicals and harmful substances that are formed during the biodegradation of the cross-linked scaffold matrix [59]. Thus, the cross-linkers, which do not jeopardize the colonization and viability of the cells in the repaired tissue, are the most optimal. Genipin has been shown to be an applicable cross-linker for tissue engineering purposes, and it has been used to cross-link chitosan to chitin, gelatin, and collagen matrixes with low cytotoxicity risks [61,62,63,64].

In summary, a wide variety of different kinds of chitosan and chitosan composite scaffolds are available for cartilage tissue engineering purposes. However, there is still a lack of proper long-term in vivo studies, which makes it difficult to evaluate the feasibility of these scaffolds and proceed to clinical human studies.

#### 2.2.3. Hyaluronan as a Cellular Scaffold

Hyaluronan (also known as hyaluronic acid) is a naturally occurring anionic and nonsulfated GAG, which is not covalently attached to a protein core [65]. Structurally, it consists of repeating units of d-glucuronic acid and *N*-acetyl-d-glucosamine, which are linked via an alternating pattern of β-1,3 and β-1,4 glycosidic linkages [66]. Hyaluronan differs from other cartilage-specific GAGs due to the lack of sulfate groups. Therefore, hyaluronan molecules are not able to form disulfide cross-links with sulfated molecules. However, glucuronic acid contains a carboxylate group, which can be used for chemical modifications and the cross-linking of hyaluronan molecules [67]. Hyaluronan is an abundant component in the articular cartilage, but it is also found in the skin, synovial fluid, and different soft tissues [65]. In the articular cartilage and synovial fluid, hyaluronan acts together with lubricin and dipalmitoyl phosphatidyl choline to lubricate the hyaline cartilage surfaces in the synovial joints [68]. In addition to viscoelasticity- and lubrication-related functions, hyaluronan can bind to the aggrecan molecules and form larger aggregate structures in the cartilage ECM [69]. Moreover, hyaluronan has a biological role in the cartilage tissue to regulate and associate with many cellular responses, such as cellular adhesion, proliferation, and differentiation [70,71].

Hyaluronan has a good biological activity to support neocartilage formation and the chondrogenic differentiation of MSCs [72,73]. MSCs, like chondrocytes, abundantly express hyaluronan synthases and hyaluronan receptor CD44, which are also important for MSC homing and adherence to the hyaluronan-containing ECM [74]. The injured articular cartilage itself also accumulates a high content of hyaluronan at the early stage of repair, therefore obviously also attracting the migrating MSCs [75].

The biological activity of hyaluronan polymer depends on its molecular weight [76]. It can be processed to different forms of scaffold materials, such as hydrogels, sponges, fibers, and nonwoven meshes [77]. The pure, natural hyaluronan scaffolds are usually mechanically weak and, therefore, different kinds of combinations of hyaluronan and the synthetic polymers have been developed. However, the synthetic modification of hyaluronan and/or the degradation products of the synthetic chemical derivatives can attenuate biologically important chondrogenic responses and cell viability [78,79]. Moreover, correct timing of the scaffold degradation process is critical because a rapid degradation might reduce the mechanical support and reduce, for example, the retention of ECM macromolecules [80]. On the other hand, a reduced degradation rate might interfere the formation and distribution of neocartilage tissue [80].

A wide variety of hyaluronan and hyaluronan composite scaffolds have been tested in vitro, but the number of therapeutically valid applications is still small. Nonwoven esterified hyaluronan derivatives, such as Hyaff 11, Hyalograft C, and Hyalofast, are examples of clinically tested and/or therapeutically used hyaluronan polymers [81,82,83,84], which have often been used in autologous cell therapies.

Hyaluronic acid benzyl ester polymer—Hyaff 11—supports the chondrocyte phenotype and normal cellular adhesion and activity, which are needed for the maintenance of the native-like hyaline cartilage [82,85]. It degrades in a few months after in vivo implantation with hyaluronan as the main degradation product [86].

Hyalograft C autograft is a Hyaff 11-based scaffold, which was used with patients’ harvested healthy chondrocytes for autologous chondrocyte implantation [87]. At first, the cells from intact marginal areas of the patient’s cartilage were isolated and expanded prior to their seeding on a three-dimensional Hyalograft C scaffold. The chondrocytes were then grown on Hyalograft C scaffold to obtain a density of four million cells per cm^2^ per graft, and the second surgical operation was made to implant the scaffold to the defected cartilage area. This method simplifies cellular implantation compared to the originally introduced technique using a periosteal flap to cover the treated site [2], and there is no need for an additional fixation of Hyalograft scaffold [88,89]. Despite the promising follow-up studies [88,89,90], actual randomized clinical studies of Hyalograft C are missing, and the manufacturer of Hyalograft C scaffolds seems to have withdrawn this product from the European market in 2013.

Hyalofast also belongs to a group of esterified hyaluronan scaffolds, and it has been designed to treat both chondral and osteochondral defects [91,92]. There have been some clinical trials using the scaffold. However, although the results have supported the beneficial nature of hyaluronan-based scaffold, the number of clinical studies based on Hyalofast is currently very limited [93]. 

Glycosil^®^ is a thiol-modified form of hyaluronan, and it can form sulfate cross-links with other thiol-modified hyaluronan molecules. Moreover, it can be covalently modified with polyethylene glycol diacrylate to form hyaluronidase degradable hydrogels [94,95]. Hystem™ is a commercial trademark of thiol-modified hyaluronan hydrogel kit, which contains Glycosil^®^, thiol-reactive cross-linker, Extralink^®^, and possible additional additives, such as denatured collagen fibers, to improve cellular adhesion properties. It offers a functional three-dimensional platform differing in pore sizes and cross-linking, and the chondrocytes seeded in it could produce a cartilage-type of tissue, which matures along the prolonged culture time [96]. However, a clear potency to improve in vitro neocartilage tissue formation has not been seen, although the embedded chondrocytes apparently have a good capacity to grow and synthesize abundant ECM in in vitro cultivated scaffolds [96]. Thus, additional studies are needed to determine whether thiol-modified hyaluronan hydrogels can be applied for cartilage repair tissue engineering in future.

#### 2.2.4. Agarose and Alginate

Agarose and alginate are both natural carbohydrates that are manufactured from marine algae, commonly known as seaweeds [97]. Both of them can be used to form hydrogels for scaffolding purposes [98,99]. Agarose polymer has a linear structure, and it consists of repeated d-galactose and 3,6-anhydro-l-galactopyranose units. Alginate polymer, on the other hand, consists of d-mannuronate and l-guluronate residues, which can form monomers in both a consecutive or alternating pattern. Agarose- and alginate-based polymers have been studied for cellular seeding of chondrocytes for tissue engineering purposes [98,99,100,101]. They are applicable to be mixed either together or with some other polymer material, such as with chitosan to form a composite material to improve mechanical strength, porosity, cellular adhesion, or some other desired feature of scaffolds [102]. Although many cell types can be encapsulated in agarose- or alginate-based hydrogels and many in vitro studies have revealed a compatibility of carbohydrate hydrogels to maintain chondrogenic or chondrocyte-specific phenotype [99,101], routine therapy-related clinical applications have not been reported.

Cartipatch^®^ (Tissue Bank of France, Lyon, France) is a clinically tested agarose–alginate scaffold, which was designed for treatments of chondral and osteochondral lesions. A clinical phase-II study with a small number of patients revealed the potency of Cartipatch^®^, but a later two-year randomized controlled trial showed that the functional outcomes of mosaicplasty led to better outcomes than Cartipatch^®^ [101,103].

## 3. Decellularized Cartilage as a Scaffold

Decellularization of the tissues has been considered as a suitable basis for scaffolds used for tissue engineering purposes as such structures basically have the correct tissue assembly of the macromolecules. Foundations for successful decellularization are that the process eliminates any possible disease transmission, diminishes or removes the components raising antigenicity or immune responses, and reduces the risk of rejection of the implanted material. Ideally, decellularization should clear away the cellular material while still preserving the original composition and the architecture of the tissue and also maintaining its mechanical properties.

The cartilage and the meniscal tissues have a massive ECM, and it is a challenge to obtain correct cellular colonization in regard to the location and penetration of cells into the deeper parts of the decellularized tissue. This is witnessed by studies that have shown that there is absent or a low infiltration of cells into an acellular porcine meniscus [104] and a tracheal cartilage [105].

Nevertheless, numerous methods have been applied to produce decellularized cartilage scaffolds. Hypotonic buffers and detergents have been exploited in order to lyse cells and solubilize cell membranes [106]. Repeated freeze–thaw cycling and pulverization has also been used to enhance the efficiency of the decellularization process [107]. The success of decellularization is understandably more efficient in smaller tissue pieces, but it still leads to impairment of the mechanical property of the tissue. To obtain better properties of engineered cartilage, freeze-sectioned acellular cartilage sheets together with expanded chondrocytes were cultured in a sandwich model in order to improve the properties of the constructs [108]. Although the Young’s moduli of the constructs were at a good level—reaching about 87% of the normal ear cartilage—the nondegraded acellular remainders were still microscopically visible [108]. Good compressive modulus was also achieved for decellularized and methacrylated cartilage ECM, which was seeded with rat bone marrow-derived MSCs and cultured for six weeks in a chondrogenic medium [109]. The creation of microchannels (diameter 400 µm) to a 1-mm-thick articular cartilage (diameter 6 mm), together with rotational culture conditions, improved cell viability and the colonization of human infrapatellar fat pad-derived stem cells and deposition of the cartilage-specific ECM [110].

## 4. Scaffold-Free Culture Systems

A scaffold-free culture method is a tissue culture system that does not rely on exogenous cell support. Its aim is to reproduce aspects similar to native fetal and postnatal tissue development and to avoid the problem of variably biodegrading scaffold materials. In this system, cells are cultured as high-density pellets or as cell sheets without a supporting preassembled matrix. The cells are expected to build their own ECM, which is further expected to have a more native-like structure. In cell sheet engineering, cells are cultivated to highly confluent monolayer cultures on top of a thermoresponsive polymer, such as poly(*N*-isopropylacrylamide) [111]. The thermoresponsive polymer can be made to change its swelling properties at biologically relevant temperatures so that at 37 °C, it is hydrophobic and allows cell attachment; when the temperature of the polymer drops below a certain threshold, it becomes hydrophilic and the cell sheet detaches without the cell–cell and cell–ECM interactions being damaged. The sheets can then be layered, rolled, or draped over substrates to form three-dimensional constructs of different geometries [112].

It has been indicated that cartilage-type of tissue can also be grown in scaffold-free culture systems. The formation of the self-assembled tissue is then not interfered by the variably biodegrading scaffold material. High-density aggregate cultures can be made using either freshly isolated [113] or frozen chondrocytes [114] to generate three-dimensional constructs without initially expanding the cells into a monolayer, which leads to risk of dedifferentiated fibroblast-like phenotype [9]. The aggregates can be formed by either rotating a cell suspension until they gather and form a mass [115] or by seeding the cells into a cell culture insert [113] or agarose wells [116,117]. The aggregate cultures have been used to create cartilage microtissues [118], and at least one commercial application exists as well [119].

Limiting the access of cells to external interactions can also form cell aggregates. The process is sometimes referred to as “self-assembly”, and it relies on nonadherent surfaces. According to the differential adhesion hypothesis—which states that the minimization of free energy drives cells to adhere to each other [120]—the self-assembly should be more similar to embryonic morphogenesis than cell sheet engineering or other aggregate culture methods. 

**Figure 3:**
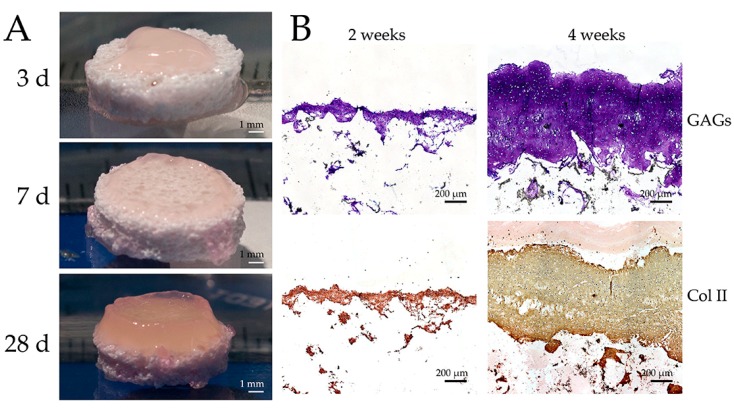
(**A**) An example of bovine articular chondrocytes (six million cells) grown inside agarose wells on top of Scaffdex Cellceram™ insert scaffold composites consisting of hydroxyapatite (60%) and β-tricalciumphosphate (40%), and cultured for three, seven, and 28 days. (**B**) The histological sections of chondrocyte/Cellceram™ inserts stained with Toluidine blue for glycosamionoglycans (GAGs) (blue color in upper images) and immunostained for the type II collagen (Col II, brownish color in lower images) after two and four weeks culture period.

Scaffold-free cultures can also be combined with bone substitutes to build biphasic constructs. An example of bovine articular chondrocytes seeded on top of Scaffex Cellceram™ insert scaffold composed of hydroxyapatite (60%) and β-tricalciumphosphate (40%) and cultured for up to four week is shown in Figure 3. A gradual growth of the construct can easily be observed, as can the abundant presence of cartilage-specific ECM molecules type II collagen and GAGs.

There are some caveats in the scaffold-free methods that need to be considered. Since there is no provided ECM to offer bulk to the construct, a high number of cells is usually required to produce appreciable tissues [114,116,117]. Similarly, as there is no ECM to guide the cells, there is often a need for mechanical and chemical stimulation of the cultures in order to guide ECM production [116,121,122]. This also means that the start of the culture can be precarious for the cell phenotype as there are no physical signals coming from the ECM. It may be necessary to wait until the cells have produced sufficient ECM before mechanical stimulation can be started. The cellularity is then modulated as the maturation of the tissue proceeds.

## 5. Bioprinting of Cartilage Tissue

The idea of being able to manufacture the structure, the ECM composition and cellular location, and the amount of one or several cell types in a fabricated tissue, or even an organ, constructs have fascinated researchers. Three-dimensional bioprinting is anticipated to be one such technique, which has become more and more widely accessible as printing technology has evolved. The prices of printing devices have also remarkably decreased since Dr. Hull presented the idea in 1986 [123]. Biomimicry, autonomous self-assembly, and microtissue-based methods are the three general approaches of three-dimensional bioprinting that have been broadly used in tissue engineering and regenerative medicine [124,125]. Inkjet, extrusion, laser-assisted, and stereolithography bioprinting are the basic techniques used, with all of them having their own advantages and disadvantages [124,125]. Scaffolds fabricated with three-dimensional printing provide the possibility of creating complex geometries, different porosities, and co-culture of different types of the cells as well as incorporation of growth factors, for instance, in microcarriers [124,125]. As the creation of vascular network is a great challenge in the manufacture of bioprinted constructs, the cartilage as an avascular tissue can, in one sense, be considered as easy to fabricate. However, the challenge of articular cartilage fabrication is the assembly of the tissue, which has the adequate mechanical properties, mainly provided by the organization of collagen and the PGs. Such an issue has obviously smaller significance, for instance, in the cartilage of trachea and ear.

Bioprinting is also of interest for cartilage tissue engineering purposes as the gradients of the cells and the ECM of the tissue constructs can be generated during the printing stage itself. There are also numerous biomaterials that can be tested as bioinks to adhere the printed cellular and ink components at the desired locations and as gradients in fabricated tissue to mimic the native structure of the cartilage. Bioprinting with multiple bioinks offers the possibility of generating osteochondral and zonally organized tissue constructs with suitable mechanical properties. The major challenge is to select materials that maintain the chondrocyte phenotype and allow maturation of the functional cartilage tissue.

### 5.1. Hydrogels

Hydrogels have been assigned as attractive materials for bioinks in three-dimensional bioprinting due to their ability to mimic the natural ECM and due to their biocompatibility, low cytotoxicity and high water content. To be suitable for three-dimensional bioprinting, hydrogels must be viscous enough to keep its shape during printing and have the cross-linking ability to retain the three-dimensional structure after printing. However, the innate viscous properties of hydrogels can make them fragile. Therefore, a combination of nanofibrillated cellulose, alginate, collagen, agarose, hyaluronan, or chitosan have been tested to create an adequate environment for the maintenance of a high level of live cells after the bioprinting [126,127,128].

#### 5.1.1. Cellulose Nanofibrils

Cellulose nanofibrils (also called microfibrillated cellulose or nanocrystalline cellulose), especially bacterial nanocellulose fibrils, have been used as a component of scaffolds in cartilage tissue engineering due to their high surface area, hydrophilicity, broad chemical modification capacity, good mechanical properties, biocompatibility, and similarity in size to collagen fibrils [126,127,128,129]. It has been reported that nanocellulose-based cellulose/alginate (80/20) bioink was successful for three-dimensionally bioprinted human ear and for sheep meniscus, with a good amount of living cells [126,127]. Three-dimensional bioprinting with nanocellulose/alginate bioink also supported the redifferentiation of human nasal chondrocytes and the cartilage–specific ECM component of the neocartilage [126]. Another bioprinting study using human nasal chondrocytes cultured alone or co-cultured with human bone marrow-derived stem cells-laden nanofibrillated cellulose/alginate bioink produced neocartilage constructs with high fidelity and good mechanical properties as well as an increased amount of type II collagen and GAGs [130].

Human-induced pluripotent stem cells co-cultured with human chondrocytes in nanocellulose/alginate (60/40) bioink showed a nice hyaline-like cartilage with type II collagen expression in the bioprinted constructs and lacked the tumorigenic Oct4 expression of the cells cultivated in induced culture medium as well as a large increase in cell numbers [131].

#### 5.1.2. Natural Scaffolds as Bioink

Alginate, collagen, and agarose have been used in cartilage tissue engineering as a promising matrix for bioinks. Yang and colleagues showed that three-dimensionally-printed scaffold of alginate/collagen I could effectively preserve the chondrocyte phenotype by suppression of the dedifferentiation of the chondrocytes [132], facilitate cell adhesion, accelerate cell proliferation, and induce the mRNA expressions of cartilage-specific genes (aggrecan, procollagen(II)α_1_ and Sox9) [132]. In addition, alginate and agarose hydrogels support hyaline cartilage tissue development the best by producing higher contents of GAGs and type II collagen compared to gelatin methacrylamide and polyethylene glycol methacrylamide, although with less printability [133].

## 6. Conclusions

This review discusses many issues related to the successful fabrication of chondral, preferably osteochondral, tissue constructs with proper presence of chondrocytes in matured tissues. The questions associated with viability and distribution of embedded cells are handled, and the feasibility of various natural and synthetic biomaterials is evaluated. Despite the availability of manifold biomaterials applicable for regenerative medicine, careful considerations of effective cellular colonization dealing with the chemical composition, porosity, cellular adhesion, and ECM assembly have to be recognized. Even scaffold-free methods need to be regarded as those systems may best enable cells to assemble a correct ECM ultrastructure immediately upon the start of manufacturing. Three-dimensional bioprinting is a novel technology that is emerging as the prices of printers get cheaper. However, special attention has to be paid on finding the best suitable bioinks.

Although prostheses normally provide good functionality of joints after reconstruction surgery, they still have a limited life span. In an ageing population, it can be expected that the need for alternatives to prostheses will increase. Therefore, efforts to advance biological, cell-based repair methods are warranted. Experiences collected on features of the used biomaterials can also be considered useful for researchers working on other fields as many biomaterials may give solutions to their regenerative needs as well.

It has been shown that there are numerous feasible approaches aimed at fabrication of native-like articular cartilage, and it is obvious that there will be no single way to succeed in it. The most critical challenge is the way the protocols manage to assemble the ECM and chondrocytes so that the tissue-engineered cartilage has the stiffness and the structural architecture that are adequate to withstand and cushion the mechanical loads present at articulating joints. These cell-based methods must pay special attention to how the cellular colonization is arranged in the best way to enable cells to stay viable and perform the differentiation and maturation in an efficient way to assemble the tissue that are durable and functionally relevant.

## Abbreviations

ECM	Extracellular matrix
GAGs	Glycosamionoglycans
MSC	Mesenchymal stem cell
PG	Proteoglycan
